# Measuring experiences of facility-based care for pregnant women and newborns: a scoping review

**DOI:** 10.1136/bmjgh-2020-003368

**Published:** 2020-11-20

**Authors:** Elysia Larson, Jigyasa Sharma, Khalidha Nasiri, Meghan A Bohren, Özge Tunçalp

**Affiliations:** 1 Department of Obstetrics and Gynecology, Beth Israel Deaconess Medical Center, Boston, Massachusetts, USA; 2 Global Health and Population, Harvard T.H. Chan School of Public Health, Boston, Massachusetts, USA; 3 Schulich School of Medicine and Dentistry, London, Ontario, Canada; 4 Department of Sexual and Reproductive Health and Research, including UNDP/UNFPA/UNICEF/WHO/World Bank Special Programme of Research, Development and Research Training in Human Reproduction (HRP), WHO, Geneve, Switzerland; 5 Centre for Health Equity, University of Melbourne School of Population and Global Health, Melbourne, Victoria, Australia

**Keywords:** systematic review, health services research, maternal health

## Abstract

**Background:**

Access to high-quality, person-centred care during pregnancy and childbirth is a global priority. Positive experience of care is key in particular, because it is both a fundamental right and can influence health outcomes and future healthcare utilisation. Despite its importance for accountability and action, systematic guidance on measuring experience of care is limited.

**Methods:**

We conducted a scoping review of published literature to identify measures/instruments for experience of facility-based pregnancy and childbirth (abortion, antenatal, intrapartum, postnatal and newborn) care. We systematically searched five bibliographic databases from 1 January 2007 through 1 February 2019. Using a predefined evidence template, we extracted data on study design, data collection method, study population and care type as reported in primary quantitative articles. We report results narratively.

**Results:**

We retrieved 16 528 unique citations, including 171 eligible articles representing, 157 unique instruments and 144 unique parent instruments across 56 countries. Half of the articles (90/171) did not use a validated instrument. While 82% (n=141) of articles reported on labour and childbirth care, only one reported on early pregnancy/abortion care. The most commonly reported sub-domains of user experience were communication (84%, 132/157) and respect and dignity (71%, 111/157). The primary purpose of most papers was measurement (70%, 119/171), largely through cross-sectional surveys.

**Conclusion:**

There are alarming gaps in measurement of user experience for abortion, antenatal, postnatal and newborn care, including lack of validated instruments to measure the effects of interventions and policies on user experience.

**Protocol registration details:**

This review was registered and published on PROSPERO (CRD42017070867). PROSPERO is an international database of prospectively registered systematic reviews in health and social care.

**Key questions**

**What is already known?**

- Positive experience of care is an essential aspect of quality of care: it is both a fundamental right and it can influence health outcomes and future healthcare utilisation.
- Yet, there is evidence from multiple countries that 20% to 42% of people are mistreated during childbirth, a particularly egregious type of poor user experience.
- To our knowledge there is one published systematic review that assesses validated measures for user experience during childbirth, and at least two reviews of methods to specifically measure mistreatment during childbirth.

**What are the new findings?**

- We included 171 articles from 56 countries globally.
- There are limited articles assessing how programmes or policies affect user experience and few that look at how user experience changes over time.
- Inequalities between and within different groups (such as adolescents, migrants, individuals with disabilities, minorities) are understudied. Further, there is extremely limited literature on user experience during abortion and newborn care.

**What do the new findings imply?**

- Many instruments exist for user experience during pregnancy, childbirth and postnatal periods and these instruments need to be consolidated, validated and expanded based on the purpose of the research, programme or accountability mechanism.
- Future research should apply these instruments to under-represented and under-served populations like adolescents and birthing people who are unmarried and across under measured areas in the care continuum, including abortion and newborn care.
- Positive experience of care is not a luxury, but a necessity; and therefore, as efforts to improve quality of care in low- and middle-income countries advance, they should include efforts to measure and improve experience of care as well.

## Introduction

More people than ever before are going to facilities to receive healthcare during pregnancy, childbirth and postpartum. However, quality of care remains substandard globally: facility infrastructure is lacking, the provision of care fails to meet evidence-based standards and birthing people and their newborns are subject to mistreatment and neglect.^1–4^ Poor quality of clinical care directly affects maternal morbidity and mortality and impedes the achievement of the Sustainable Development Goals by 2030.^5–9^ Furthermore, poor user experiences violate birthing people’s rights to be treated with respect and dignity and can negatively affect their health outcomes and future health-seeking behaviours.^1 10 11^

The WHO defines experience of care for pregnant people and newborns along three components: (1) effective communication; (2) respect and dignity; and (3) emotional support, and postulates a bidirectional relationship between experience and provision of care in determining key person-centred and health outcomes.^12^ The recent Lancet Global Health Commission on High Quality Health Systems^13^ articulates an additional ‘user focus’ component, and the report ‘Delivering quality health services; a global imperative for universal health coverage’ highlights quality that is ‘people-centred’.^14^ These definitions of experience of care illustrate the salience of user experience as an integral component of high quality care. However, despite theoretical advancements, there has been inadequate empirical work on assessing the level of, and improving, experience of care.^15^

Appropriately measuring user experience is critical for both accountability and action.^13^ However, because systematic guidance on measuring user experience is limited, it is likely that a diverse set of indicators and measurement methods are currently being used in maternal and newborn health. While recent reviews have focussed on measurement of one aspect of user experience in maternal health, mistreatment in childbirth,^1 16 17^ to our knowledge, there is only one systematic review reporting on quantitative instruments for measuring people’s childbirth experience,^18^ and that review was limited to validated instruments.

In this context, we conducted a scoping review of measures and instruments currently in use globally to quantitatively assess experience of facility-based care for pregnant woman and newborns. More specifically, we aim to identify indicators and instruments across the four components of user experience as defined by the WHO and the Lancet Global Health Commission on High Quality Health Systems in the Sustainable Development Goal Era (HQSS), in order to inform future research, monitoring and implementation. This review is meant to provide a starting point for others who are seeking instruments to measure user experience and identify current gaps in measurement for research, action and accountability.

## Methods

### Search strategy and selection criteria

This scoping review focusses on indicators and instruments used to measure one broad domain of person-centred care: user experience (box 1). User experience indicators focus on people’s interactions with healthcare providers and the healthcare system. Recognising the need to distinguish between user experience and user satisfaction,^19^ we began with a conceptual framework for user experience that is adapted from the WHO Quality of Care Framework for maternal and newborn health^20^ and the Lancet Global Health Commission High Quality Health System framework.^13^ This led to four domains and 13 subdomains: (1) respect and dignity (respect and dignity, privacy, non-discrimination, autonomy, confidentiality, kindness), (2) effective communication (communication), (3) support (social and emotional support) and (4) user-centred health systems (user voice, affordability, choice of provider, appropriate wait times, ease of use of the system).

Box 1 **A note on terminology**

Throughout the introduction and discussion of this paper we have chosen to use the term ‘birthing people’. This is to recognise that not all individuals who get pregnant or go through childbirth are cisgender women, who were born and identify as female. In the methods and results we use the term ‘women’ as the literature we were scoping referenced women and thus likely largely represented women.^34^ This in of itself may be a limitation in the field—that research is focussed on women and the experiences of transgender men and non-binary people who deliver may be missed in many of these studies.

We have also opted to use the term ‘user experience’ to describe an aspect of quality of care that is often referred to as ‘patient experience’ or ‘interpersonal care’. We have opted for this term in order to use inclusive terminology and not over-medicalise childbirth.^35^

The primary inclusion criterion was articles that measured at least one of the above subdomains. Additional inclusion criteria were: articles published on or after 1 January 2007, original research (ie, not an editorial, comment or newspaper article), study participants are women who are/were pregnant and/or newborns, study reports on facility-based care for pregnant or postpartum women or newborns and results include those from a quantitative research study of any design. We note that the PROSPERO registration refers to ‘pregnant women and newborns’, which reflects the language of the WHO quality of care framework.^20^ The WHO framework and this review include postpartum care, and as such we explicitly included postpartum period as part of the review. No language restrictions were imposed. We excluded articles where the only indicators of person-centred care were satisfaction with aspects of care, as satisfaction reflects a user’s evaluation of the care received rather than their report of said care, and is affected by users’ expectations.^19^

A scoping review was conducted in accordance with the Preferred Reporting Items for Systematic Reviews and Meta-Analyses extension for Scoping Reviews (PRISMA-ScR) guidelines.^21^ We searched five databases (PubMed, Embase, CINAHL, Web of Science and Global Index Medicus). Search terms were developed through consensus between authors (JS, EL, MAB and ÖT) and a research librarian was consulted to define search strategy to identify all articles measuring user experience of care for maternal and newborn health. The complete search terms used in PubMed can be found in online supplemental appendix 1. The content terms included, but were not limited to, maternal health, patient-centred care, experience, satisfaction, support, provider choice, wait time, affordability, dignity, respect, privacy, confidentiality, discrimination, communication, abuse, mistreatment and perception. The search string was modified and adapted for use in all other databases. The initial search was conducted on 15 August 2017 and updated on 1 February 2019. We supplemented the database search with a bibliography search of key articles^17 18^ to identify additional relevant articles. Trial registries and data from unpublished articles were not included. Duplicate records were deleted first using the software (EndNote) and manually if any identified later.

10.1136/bmjgh-2020-003368.supp1 Supplementary data

Four researchers (MAB, EL, JS and ÖT) conducted abstract screening. Three researchers (EL, KN and JS) subsequently reviewed full-text articles and extracted data using a standardised form developed for this review. For each step (title/abstract review, full-text review and data extraction), only one reviewer independently reviewed each paper. However, to ensure consistency across different data extractors, prior to the full-text review, each researcher reviewed the same three articles as another researcher. Any discrepancies were discussed until consensus was reached. We extracted data on study design, data collection methods, study population, timing and care type and data collection instruments and indicator domains. The full abstraction tool and resulting data are available in the online supplemental appendix 2. During the review process at BMJ Global Health, insightful reviewers asked us to abstract two additional pieces of information from the included papers: if another form of quality of care was assessed and if representatives of the study population were involved in instrument creation or use. We looked at these variables for a random subset of articles (102). For manuscripts published in a language other than English, a co-author fluent in that language reviewed the manuscript. If none of the co-authors were fluent in the language of publication, then one of the researchers worked with a colleague at the WHO to review the article together. The study protocol was registered and published on PROSPERO (CRD42017070867, https://www.crd.york.ac.uk/prospero/display_record.php?RecordID=70867).

10.1136/bmjgh-2020-003368.supp2 Supplementary data

### Data synthesis

Data were abstracted using the mobile data collection platform SurveyCTO Dobility, Inc 2020 and exported to Stata V.14 for synthesis and analysis. Data were cleaned and categorised. We grouped manuscripts by the measures and/or instrument they used, since not all measures are instruments and not all instruments are used consistently across different articles. For example, six articles reported using the ReproQ instrument and are grouped in online supplemental appendix 3. Where the articles in a group report on using the same or similar questions from the instrument, resulting in the same subdomains of user experience represented, we only count the instrument once in the numerator and denominator of the report of subdomains. Where the articles differ in the parts of the instrument used, resulting in different subdomains of user experience represented, we maintain each article as a unique contribution to the description of the representation of user experience subdomains in the literature. So one ‘unique parent instrument’ may result in two ‘unique instruments’ resulting in two articles each.

10.1136/bmjgh-2020-003368.supp3 Supplementary data

We report summary statistics describing the aims, methods of data collection and domains of user experience. For each included article, the reported aims were assigned one of the following categories: instrument validation, measurement (eg, prevalence, determine correlates of user experience), evaluation (eg, of programme or policy) or measurement of a domain other than user experience (eg, utilisation). We further disaggregate by year of publication (published in 2007 to 2015 vs 2016 to 2019). The year of 2015 was determined as an appropriate cut-off, because it was the beginning of the Sustainable Development Goal Era which emphasised the importance of quality care and also the year the WHO published their ‘vision’ for the quality of care for pregnant women and newborns.^20^ We report geographical variation through a heat map by country and again by frequency of publication for each World Bank designated country-income group.

We did not assess quality or risk of bias for the included articles as the objective of this review was to scope and describe the breadth of instruments and indicators used to measure experience of care and was not concerned with the magnitude or directionality of bias in any outcome variable.

This review is reported following the PRISMA-ScR statement guideline to enhance transparency in reporting scoping reviews.^21^ The corresponding author had full access to all the data in the study and had final responsibility for the decision to submit for publication.

#### Patient and public involvement

This study specifically addresses measurement of user experience and thus the research question was informed by literature on patient, or user priorities, experiences and preferences. Patients or the public were not, however, directly involved in the design, or conduct, or reporting, or dissemination plans for this scoping review. Data were not collected directly from patients for the purposes of this research.

## Results

**Figure 1:**
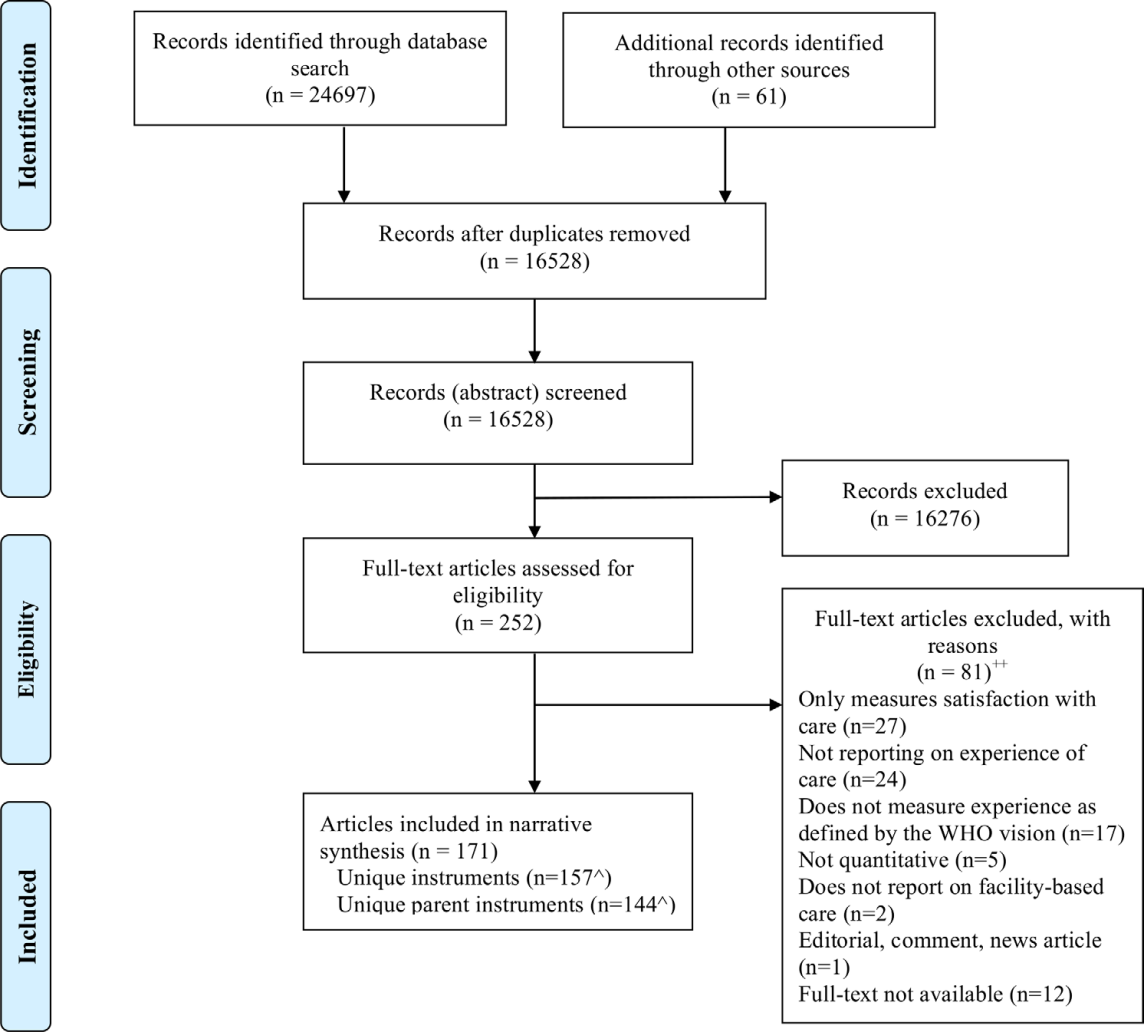
PRISMA (PreferredReporting Items for Systematic Reviews and Meta-Analyses) Flow Diagram. ++ Articles could be excluded for more than one reason.ˆEach article contributed one main instrument toward this count.

A total of 24 697 records were identified through the database search. An additional 61 were identified through additional search methods (figure 1). Of these, 171 records met eligibility criteria and were included in the narrative synthesis. Authors, titles and publication descriptions are available in online supplemental appendix 3.

The stated primary aim for more than two-thirds of the articles fell into the category of measurement (eg, prevalence or determining correlates of user experience) and only 9% (15/171) of articles aimed to evaluate programmes or policies.

**Figure 2:**
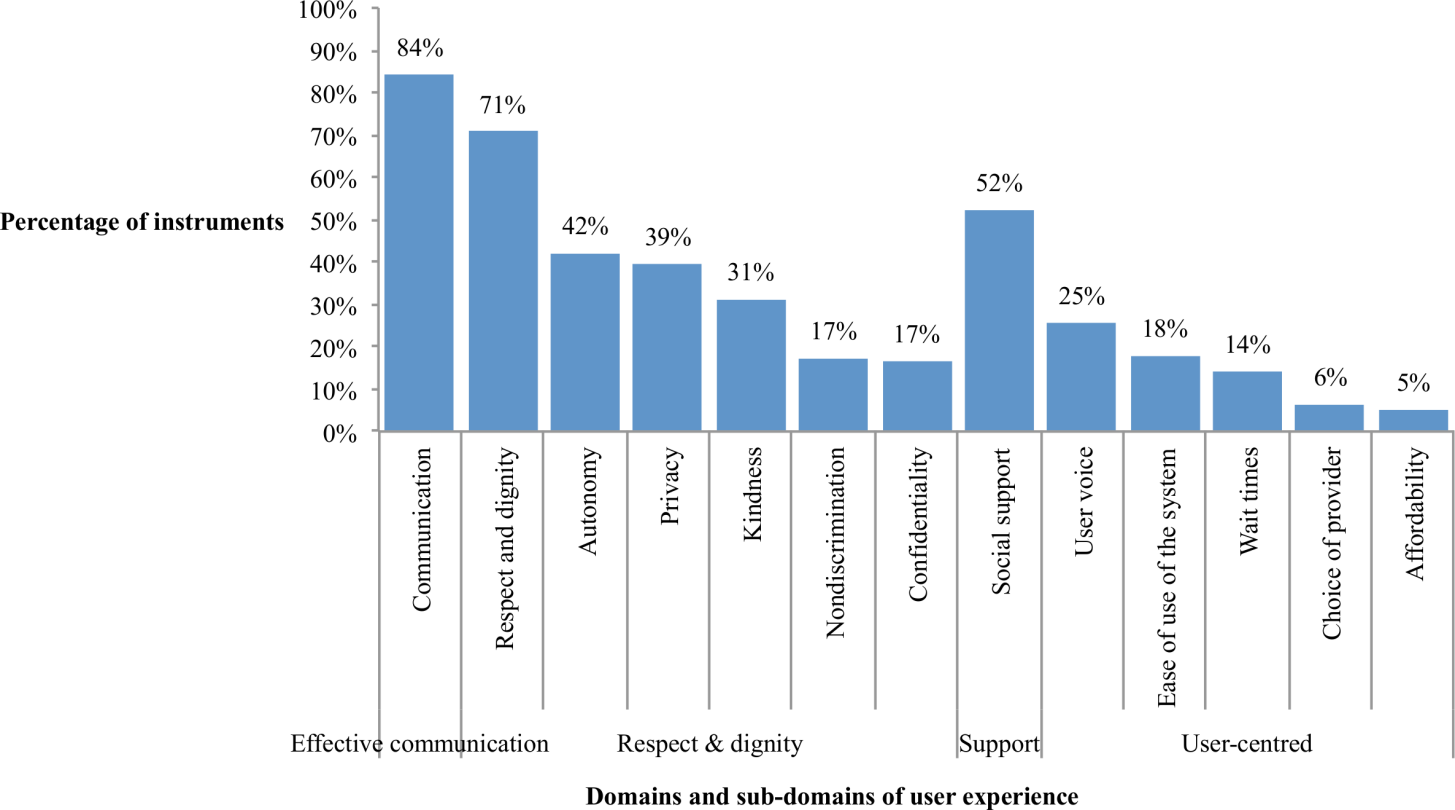
Percentage of identified measures and instruments reporting by domain and subdomain of user experience (n=157).

In half of the articles (50%), the authors did not specify a clear conceptual framework for their choice of user experience domains. The most frequently cited frameworks included the WHO Quality of Care framework^20^ and Valentine *et al*’s work on the responsiveness of health systems.^22^ Other commonly cited publications included two on mistreatment during childbirth (Bowser and Hill^23^ and Bohren *et al*^1^) and Donabedian’s framework for quality of care.^24^ The most commonly reported domains were ‘respect and dignity’ in 83% (130/157) of instruments and ‘communication’ in 84% (132/157) of instruments (figure 2). Of the 13 subdomains we assessed, the median number of domains reported on was four. Two-thirds of articles (66/102) assessed an additional form of quality, such as aspects of structural quality or indicators of competent care.

**Figure 3:**
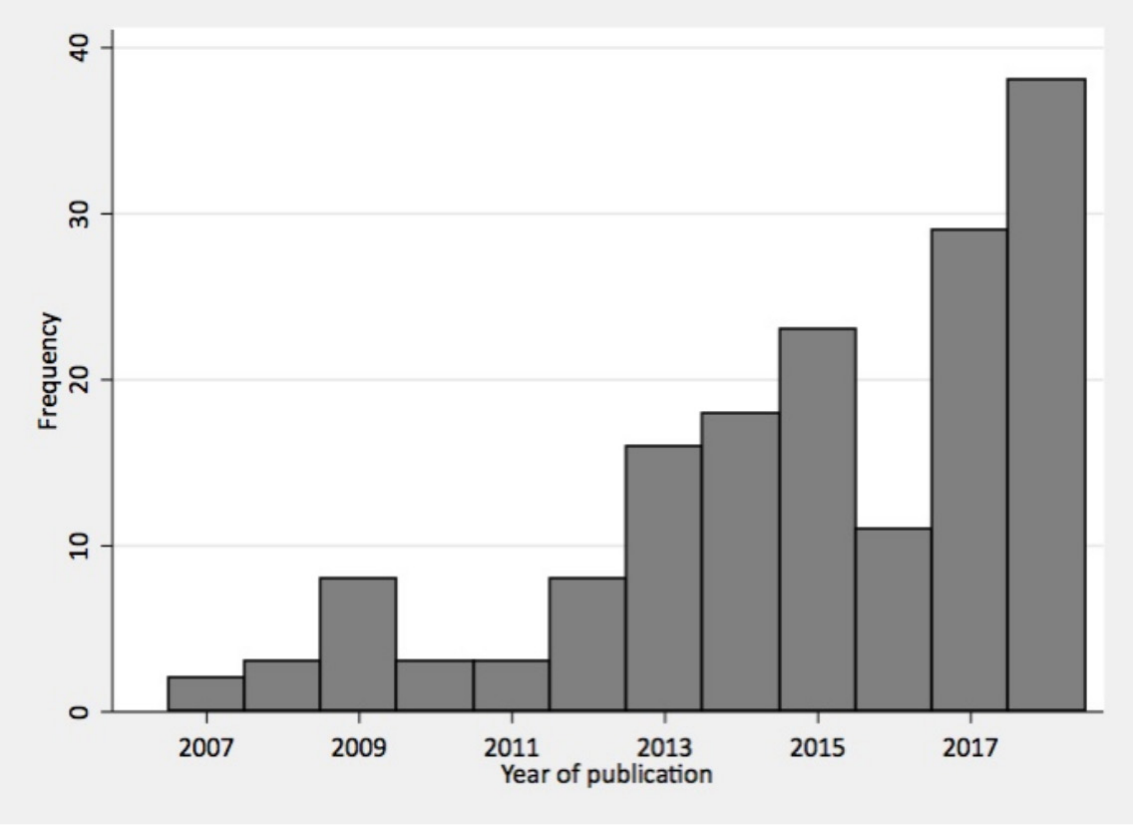
Number of articles by year^+^ of publication. +An additional nine articles from 2019 were not included in the graph, because we did not include all months from 2019 in the search.

**Figure 4:**
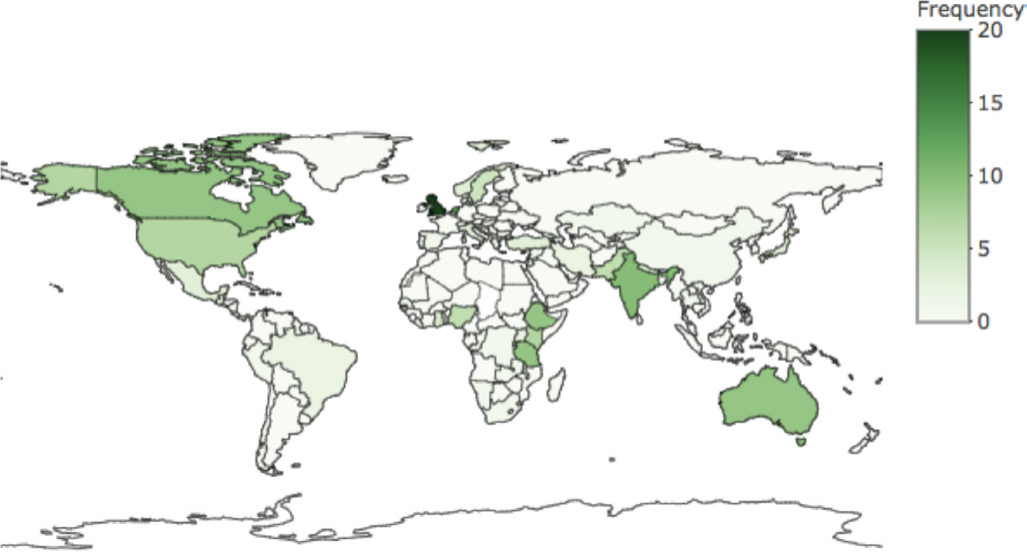
Distribution of articles by country.

The number of articles per year reporting on user experience increased from 2 in 2007 to 38 in 2018 (figure 3). Most of the articles assessed user experience during labour and childbirth (82%, 141/171) with only one study reporting on early pregnancy or abortion care (table 1). More than one-fourth of articles (44/165) excluded women with stillbirths and 41/165 excluded women with high-risk births and/or complications. Europe had the largest representation in articles (by source of data collection); the number of articles using data collection from sub-Saharan Africa increased the most from the 2007 to 2015 to 2016 to 2019 period (from 16 articles to 33 articles) (figure 4).

**Table 1:** Summary of articles included in the final analysis (n=171)

	Published 2007 to 2015	Published 2016 to 2019	Total
	N (%)	N (%)	N (%)
**Purpose**			
Main study aim			
Instrument validation	22 (26.2)	12 (13.8)	34 (19.9)
Measurement*	54 (64.3)	65 (74.7)	119 (69.6)
Evaluation (eg, of programme or policy)	7 (8.3)	8 (9.2)	15 (8.8)
Other†	1 (1.2)	2 (2.3)	3 (1.8)
**Participants**			
Number of study participants‡	430 (21 to 26 325)	875 (25 to 20 094)	585 (21 to 26 325)
Timing in continuum of care			
Early pregnancy and/or abortion	0 (0)	1 (1.1)	1 (0.6)
Antenatal care	36 (42.9)	28 (32.2)	64 (37.4)
Labour and childbirth	63 (75.0)	78 (89.7)	141 (82.5)
Postnatal care	21 (25.0)	21 (24.1)	42 (24.6)
Newborn care	2 (2.4)	10 (11.5)	12 (7.0)
Unclear	7 (8.3)	0 (0)	7 (4.1)
Location: country income status§			
Low income	9 (10.7)	23 (26.4)	32 (18.7)
Lower middle income	14 (16.7)	21 (24.1)	35 (20.5)
Upper middle income	9 (10.7)	13 (14.9)	22 (12.9)
High income	52 (61.9)	30 (34.5)	82 (48.0)
**Data collection methods**			
Reported validation			
Validation study	22 (26.2)	9 (10.3)	31 (18.1)
Used validated instrument	17 (20.2)	16 (18.4)	33 (19.3)
Has components of validated instrument	13 (15.5)	4 (4.6)	17 (9.9)
Instrument not validated	32 (38.1)	58 (66.7)	90 (52.6)
Timing¶			
During facility stay or immediately after discharge	25 (29.8)	29 (33.3)	54 (31.6)
Within 1 week	11 (13.1)	6 (6.9)	17 (9.9)
8 days to 6 weeks	7 (8.3)	5 (5.7)	12 (7.0)
7 weeks to 1 year	25 (29.8)	29 (33.3)	54 (31.6)
More than 1 year	4 (4.8)	12 (13.8)	16 (9.4)
Unclear	12 (14.3)	6 (6.9)	18 (10.5)
Total number of articles	84	87	171

*For example, measuring prevalence of aspects of user experience and/or determining correlates of user experience.

†The primary aim of these articles was to measure something other than user experience (eg, utilisation).

‡Median (range).

§World Bank country income status at the time of publication.

¶After delivery in the case of childbirth, or date of services rendered in the case of outpatient care.

Almost all articles included data collected through a self-administered (47%, 80/171) and/or interviewer-administered (52%, 89/171) survey. Observations were conducted in 8% (13/171) of articles. Almost all of the articles (91%, 155/171) were cross-sectional and only 5% (8/171) were longitudinal or cohort studies. Most (11%, 18/171) of the articles used data from primary research studies rather than from large-scale surveys (for example, regionally representative data sets such as the ‘Having a Baby in Queensland survey’ or multinational data sets such as the Service Provision Assessments).

More than half (53%, 90/171) of studies did not use a validated instrument and/or validation was not an objective of the study. Most articles reported using one measure/instrument (89%, 153/171) and the primary measure/instrument had a median of 21 questions (range 1, 200). Only 18% (18/102) of articles clearly report that they used feedback from the study population (usually through preliminary in-depth interviews or focus group discussions) in the process of developing or choosing their instruments. Others may have done the same, but did not explicitly state it in their methods section.

## Discussion

This scoping review included 171 articles reporting on aspects of user experience of pregnant women and newborns during the perinatal period. We identified an increase in articles over the past 12 years, likely reflecting an increased global interest in quality of care generally, and user experience specifically. This review aimed to give a comprehensive review of the current state of measures and instruments used in research on user experience and can be used to guide researchers and implementers on both available instruments and gaps in area of study.

The primary aim of most of the articles was to describe the state of user experience. From these descriptive articles we know that user experience is often suboptimal and that some groups (eg, adolescents, migrants, individuals with disabilities and minorities) have worse experiences than others.^4 25^ However, very few articles included in our review had as their main aim the evaluation of programmes or policies that may be designed to address/mitigate the gaps in user experience. Furthermore, few articles reported on user experience longitudinally, either through a cohort study or repeat cross sections. This focus on a single episode of care leads to a limited understanding of how experiences at one point may affect decision-making and health of the individual, how perceptions or experiences may change over time (such as throughout a pregnancy, or at different time points between the time of birth and throughout the postpartum period) or which policies or programmes could be most effective in its improvement. For instance, poor experience of antenatal care may influence a woman’s choice of facility or provider, or in absence of options, decision to forgo facility-based childbirth care altogether.^10^

There was no single, comprehensive, validated instrument for measuring all aspects of user experience. Therefore, while the research in this area is exploding, comparability is limited—only four articles reported on data from multiple countries and only 11% used data from large-scale surveys. Notably, more than half of papers included in this review were based on instruments that have not been validated. Others used instruments that were adapted from validated instruments, meaning they are no longer valid. We identified 45 unique, validated instruments measuring various domains of experience of care. Lack of validated comprehensive instruments for measuring all domains could partly explain this phenomenon of multiple instruments. Failure to use validated instruments even when the option exists limits researchers’ ability to conduct comparative studies across populations, contexts and time. It also suggests that subjectivity and appropriateness of the tool may not have been addressed.^19^ Additionally, we observe a geographical and time trend in use of validated instruments: prior to 2015, most studies were conducted in high-income country settings and a higher proportion among them employed validated instruments or were validation studies, whereas post-2015, despite the increase in studies in low- and middle-income settings, only a small proportion of studies used validated instruments. While this indicates the possibility for an expanded use of validated instruments, it is also important to note that when quantitative instruments are translated between languages and cultures, even validated tools may require additional work such as cognitive interviewing to ensure data quality, cultural appropriateness of measures and the validity of findings.^26^ This review highlights a pressing need for developing, or using if it already exists, validated instruments for measuring various domains of experience of care. The importance of developing a coordinated approach to appraising and communicating available evidence on better measurement in global maternal and newborn health has been discussed elsewhere,^27^ our review, documenting the widespread use of multiple, non-validated instruments, provides further evidence to support this call to action.

The timing of data collection for these studies was varied, with about one-third of the studies collecting data during the users’ stay or immediately on exit, and most of the remaining occurring several days to 1 year after the point of care. There are advantages and disadvantages to both measuring close to the receipt of care and a while after care. Immediately after a person receives care, they may feel a sense of relief (eg, in the case where they are bringing home a new, healthy baby), despair (eg, in the case where they have just received a terminal diagnosis) or anything in between, affecting how they interpret the care received. The review of methods for measuring prevalence of disrespect and abuse during childbirth by Sando and colleagues gives a nice discussion of the tradeoffs, including risk of courtesy bias when assessed close to the receipt of care, and risk of priming (the individual has more time to think about their care and be primed by other experiences or questions to think of it as more or less favourably), recall bias and lower response rates at later time points.^17^ Recognising this trade-off, and in absence of a perfect, reference measure, one must consider methodological rigour together with logistical constraints and weigh each of these considerations in their interpretation of the indicators obtained. For example, facility exit surveys may be more feasible for routine quality improvement efforts given that community follow-up can be resource intensive. However, facility exit surveys are conducted close to the time of care and typically within or close to the location of care, which may affect the participant responses in two key ways: (1) less likely to report negative experiences; and (2) less time to process and reflect on the care received.

An additional source of potential bias in many of the studies comes from the participant inclusion/exclusion criteria. Who we measure user experience for matters. One in four manuscripts excluded women with stillbirths and one in four excluded women with high-risk births or complications. In addition, as described in the box, this review and the article in it do not explicitly stratify by gender. These people may have different experiences of care; in one of the reviewed papers where high-risk people were included, they perceived quality and responsiveness as higher than people with a healthy birth.^28^ Systematic exclusion of a subset of the population from studies translates into a non-generalisable sample, with any measure of experience of care thus derived not representative of all pregnant people. Furthermore, lack of evidence on experience of pregnant people across the spectrum of risk will mean that any policies that are based on available evidence will fail to address the unique needs, if any, of the high risk population subset.

This scoping review had some limitations. First, categorisation of instruments into different domains and subdomains was subjective. Operational definitions were lacking in many articles and, where available, were not consistent across articles. Therefore misclassification across categories is possible. In addition, in the case of at least communication, there may be some overlap between user experience and competent care. For example, while a provider asking about symptoms is a form of communication, it is directly related to her provision of competent care. One framework disaggregates care between interpersonal and informative care,^29^ touching on the potential overlap communication may play over the two broad areas of quality of care. Second, 17 articles were excluded for not measuring user experience as defined our framework, which merged the WHO vision and HQSS framework.^13 20^ We may be missing an area of care experience that some people consider an important aspect of user experience. However, given that the frameworks used were based on prior evidence and contain broad categories, it is unlikely that major areas were missed. Third, in this review we did not assess community participation in the design, implementation or receipt of funding of these studies. In order to assess and achieve equity in user experience, research must be done with cultural rigour, otherwise, as noted by Scott, Bray and McLemore, results may lack “clarity and cultural relevance to community identified research priorities”.^30^ Finally, the terms used in identifying the articles were selected to ensure comprehensiveness and precision of the search; despite efforts to reduce such occurrence, we could have missed some relevant articles that did not mention any of the terms included in the search string.

This scoping review also has several strengths. First, the review includes articles that include both validated and non-validated measures and/or instruments for user experience, allowing us to review a broad scope of what is functionally being used in measurement. Second, the review included literature from both high income and low- and middle-income countries without a language restriction, creating a comprehensive mapping of current state of experience of care measurement to identify gaps and inform future research. Finally, this review assessed measures and/or instruments across the spectrum of care from pregnancy to postpartum, including abortion care, which is an important but often neglected aspect of reproductive healthcare.

Given these findings, there are clear implications for future research. First, instruments exist for user experience during pregnancy, childbirth and postnatal periods and these instruments need to be consolidated, validated and expanded based on the purpose of the research, programme or accountability mechanism. The consistent reporting of conceptual frameworks and processes used to identify domains including operational definitions will be important to analyse and interpret the findings across studies. The next step in understanding the current state of user experience is to use similar instruments across multiple populations. This could be accomplished by beginning with one (or more) of the validated instruments identified in this review adapting it as needed to cover the full range of user experience and be validated within the countries under study, and then adding the instrument to one of the large-scale surveys, such as the Service Provision Assessment, Demographic and Health Surveys, WHO multi-country surveys, which would enable harmonisation across such tools reducing measurement burden.^31^ The same, or tailored versions of these instruments could also be used for quality improvement and evaluation purposes. The process of identifying and using comparable instruments should take into consideration the study purpose and how both validity and subjectivity will be addressed.^19^ Second, future research needs to adapt and apply these instruments to populations marginalised by systems of power, such as Black and Indigenous populations, people from migrant and refugee backgrounds, adolescents and birthing people who are unmarried. Using participatory methods to engage with these communities is essential to ensure evaluations of user experiences are inclusive of and responsive to cultural practices.^30^ Similarly, as the review points, despite the growing number of studies conducted in low- and middle-income countries (LMICs), measurement of user experience appears concentrated in high-income settings. Positive experience is not a luxury, but a necessity; and therefore, as efforts to improve quality of care in LMICs advance, they should include efforts to measure and improve experience of care as well. Third, instruments need to be assessed for their validity in capturing experience of care across the continuum, particularly in currently under-measured areas such as during abortion and newborn care.^32 33^

## Conclusion

There are a growing number of articles that assess user experience during the maternal and perinatal period using different measures and instruments. From our review we found that most papers were descriptive. Future descriptive work should target larger and more diverse populations, for example, through incorporating validated instruments into large-scale surveys and focussing on under-represented populations, such as people having abortions, minority groups and adolescents. Few studies measured how user experience changes over time, demonstrating a need to measure user experience longitudinally and assess how programmes and policies can affect user experience.
