# Supplementary material for: Measuring experiences of facility-based care for pregnant women and newborns: a scoping review
Source: BMJ Glob Health. 2020 Nov 20;5(11):e003368. doi: 10.1136/bmjgh-2020-003368 (PMC7682195; doi:10.1136/bmjgh-2020-003368)
Supplement: Supplementary data [file bmjgh-2020-003368supp001.pdf]

(maternal health[tiab] OR maternal service[tiab] OR maternity care[tiab] OR maternal care[tiab] OR maternity service*[tiab] OR "Maternal Health"[mesh] OR "Maternal Health Services"[mesh])*

AND

(experience[tiab] OR experiences[tiab] OR patient-centered[tiab] OR woman centered[tiab] OR women centered[tiab] OR client centered[tiab] OR satisfaction[tiab] OR social support[tiab] OR emotional support*[tiab] OR provider choice[tiab] OR choice of provider[tiab] OR wait time*[tiab] OR affordability[tiab] OR dignity[tiab] OR respect[tiab] OR privacy[tiab] OR confidentiality[tiab] OR discrimination[tiab] OR communication[tiab] OR disrespect[tiab] OR abuse[tiab] OR mistreatment[tiab] OR perception*[tiab])*
