# Supplementary material for: Measuring experiences of facility-based care for pregnant women and newborns: a scoping review
Source: BMJ Glob Health. 2020 Nov 20;5(11):e003368. doi: 10.1136/bmjgh-2020-003368 (PMC7682195; doi:10.1136/bmjgh-2020-003368)
Supplement: Supplementary data [file bmjgh-2020-003368supp002.pdf]

Question	Response options
IDENTIFICATION	
1. Reviewer name	
2. Date of data entry	
3. Publication type	1 Journal article<br>2 Dissertation
4. Record source	1 Database search<br>2 Hand search
5. Authors <Please list author's last name first>	
6. Title	
6a. Year of publication	
6b. Endnote citation <Copy and paste the full Endnote citation as is>	
ELIGIBILITY	
7. Is the study eligible for inclusion	1 Yes<br>0 No
7a. If not, excluded based on title/abstract or full-text review?	1 Title/abstract<br>2 Full-text review
7b. If not, reason for exclusion <Select first one that applies>	1 Is an editorial, comment, newspaper article or other form of popular media<br>2 Study participants are not women or newborns<br>3 Does not report on facility-based care for pregnant women or newborns<br>4 Does not report on the experience of facility-based care for pregnant women or newborns<br>5 Does not report on a quantitative research study<br>6 Does not measure experience of care as defined in the WHO Quality of Care framework (effective communication, respect and dignity, access to the social and emotional support of her choice) or HQSS Commission framework (choice of provider, short wait times, social support, affordability, ease of use of system, dignity, privacy, nondiscrimination, autonomy, confidentiality, clear communication, patient voice – being heard)<br>7 Only measures of experience of care is "satisfaction" with limited response options such as "satisfied with care - yes/no" or satisfaction with care - Likert scale"
SETTINGS & DEMOGRAPHICS	
8. Location of study<br><e.g. city, district, state, country>	
9. Urban or rural	1 Rural<br>2 Urban<br>3 Both<br>4 Unclear
10. Language of the manuscript	1 English<br>2 French<br>3 Spanish<br>4 Other (please specify)
11. Study population	

<Please provide succinct description, preferably as reported in the abstract>
12. Number of study participants<br><Sample size for assessment of measures related to experience of care>
STUDY DEISGN & OBJECTIVES
13. Study aims or objectives
14. Is this a validation study?<br>1 Yes<br>0 No
15. Study design <For measuring user experience>
16. Data source<br>1 Primary research data<br>2 Large scale survey (please specify on next page)
16a. (Large scale survey) please specify the survey country, name year, e.g. Kenya SPA 2010 or Nepal DHS 2014
17. Are the start and end date of data collection recorded? <Select 'yes' if at least the month and years are recorded><br>1 Yes<br>0 No
17a. Start date of the study data collection <Please enter 01 if DD not reported>
17b. End date of the study data collection <Please enter 30 if DD not reported>
18. What time period in the continuum of care is the article reporting on as it relates to experience of care?<br>Please select all that apply (other example = triage)<br>1 Early pregnancy and/or abortion<br>2 Antenatal care<br>3 Labor and childbirth care<br>4 Postnatal care<br>5 Newborn care
DATA COLLECTION TOOLS & METHODOLOGY
19. Identification of study population including selection of facilities<br><e.g. all woman living within the catchment population of select community health centers in Rajasthan, India>
20. Study participant exclusion and/or inclusion criteria<br><e.g. woman aged at least 15 years who delivered in dispensaries in Pwani region, Tanzania within 6-12 months prior to data collection were eligible for participation>
21. Data collection method<br><e.g. self-administered survey; interview (meaning interviewer administered survey); observation; facility records. Focus on measuring user experience>
22. Timing of data collection<br><e.g. upon discharge from health facility, within six months from receipt of facility-based care; record for time points when patient experience was measured>
23. Place for data collection<br><Please indicate any consideration for privacy/confidentiality of the respondent or other ethical concerns>
24. Response time, report if applicable<br><e.g. 45 minutes to complete the semi-structured survey; record for time points when patient experience was measured>
25. Is the instrument measuring overall experience of care?<br><This is asking if the study uses an index to measure experience of care. For example, an additive index of different components which the authors choose to present as an overall index of experience of care.>
26. What domains/dimensions/subscales within experience of care is the instrument measuring?<br><Please list all that apply>

1 Access to the social and emotional support of her choice<br>2 Affordability<br>3 Autonomy<br>4 Choice of provider<br>5 Communication<br>6 Confidentiality<br>7 Dignity<br>8 Ease of use of the system<br>9 Nondiscrimination<br>10 Patient voice<br>11 Privacy<br>12 Respect and dignity<br>13 Social support<br>14 Wait times<br>15 Kindness<br>16 Overall satisfaction<br>-99 Unclear or NA
27. How many instruments are used for measuring experience of care or select domains?
<Please complete 28-36 for each instrument used (repeat 27a XX times as reported in item 27)>
28. Name (and the acronym, if applicable) of the instrument
29. What is the instrument measuring? <e.g. experience of care, or a specific domain within it>
30. What is the source of information for this instrument? <e.g. woman, health records, observers>
31. Please provide a brief description of the data collectors <e.g. midwives, doctors, etc.>
32. Is the instrument validated?<br>1 Yes<br>0 No
32a. If validated, please provide the reference for validation study. Indicate NA, if citation not provided/available.
32b. If the instrument is not validated, does it have some components of a validated tool?<br>1 Yes<br>0 No
32bi. If "yes" in 32.b, please briefly describe/list the validated tool used.
32bc. Please provide the reference for validation study. Indicate NA, if citation not provided/available.
33. Do the authors describe theoretical/conceptual frameworks underpinning the instrument?<br>1 Yes<br>0 No
33a. Please briefly describe/list the theoretical framework used.
34. Number of items <e.g. number of questions or observation categories>
35. Is the instrument available?<br>1 Yes<br>0 No
35a. Please list the questions asked or items assessed by this instrument.
36. What is the response scale?<br>Select all that apply:<br>1 Likert scale - 3 point<br>2 Likert scale - 4 point<br>3 Likert scale - 5 point<br>4 Multiple choice options<br>5 Yes/no<br>6 Other (specify)
6. OUTCOMES

37. What is the response rate reported by the authors?<br><Please record percentage, e.g. 97.5 for 97.5%. Indicate -99 if not provided>
38. Briefly describe the main findings on experience of care related outcomes reported in this study.<br><e.g. prevalence of disrespect and abuse>
39. Is any association investigated between experience of care (or a domain within it) and other covariates (ex. demographic predictors, or health outcomes)?<br>1 Yes<br>0 No
39a. Please report the covariates.
METHODOLOGICAL REMARKS
40. What were the limitations that the authors discussed regarding measuring experience of care or its domains?
41. What were the strengths that the authors regarding their approach to measuring experience of care or its domains?
REVIEWER COMMENTS
42. Did you use any additional references from the record to complete the extraction form?<br>1 Yes<br>0 No
42a. Please provide the citation used to complete this form
43. Please briefly describe any additional remarks you have regarding measurement of experience of care reported in this record.
44. Any additional reviewer comments?
