# Supplementary material for: Measuring experiences of facility-based care for pregnant women and newborns: a scoping review
Source: BMJ Glob Health. 2020 Nov 20;5(11):e003368. doi: 10.1136/bmjgh-2020-003368 (PMC7682195; doi:10.1136/bmjgh-2020-003368)
Supplement: Supplementary data [file bmjgh-2020-003368supp003.pdf]

First author	Title	Year of public-ation	Main study aim	Country of data collection	Period of care	Instrument validation	Domains
Freedman	Eye of the beholder? Observation versus self-report in the measurement of disrespect and abuse during facility-based childbirth	2018	Instrument validation	Tanzania	labor and childbirth	Instrument not validated	communication, respect & dignity, privacy, nondiscrimination, social support, affordability
Kruk	Disrespectful and abusive treatment during facility delivery in Tanzania: a facility and community survey	2018	Measurement	Tanzania	labor and childbirth	Instrument not validated	communication, respect & dignity, autonomy, privacy, confidentiality
Kujawski	Community and health system intervention to reduce disrespect and abuse during childbirth in Tanga Region, Tanzania: A comparative before-and-after study	2017	Program/Policy evaluation	Tanzania	labor and childbirth	Instrument not validated	communication, respect & dignity, autonomy, privacy, confidentiality, social support
Kujawski	Association Between Disrespect and Abuse During Childbirth and Women's Confidence in Health Facilities in Tanzania	2015	Measurement	Tanzania	labor and childbirth	Used validated instrument	respect & dignity
Kingston	Comparison of adolescent, young adult, and adult women's maternity experiences and practices	2012	Measurement	Canada	antenatal, labor and childbirth	Instrument not validated	communication, respect & dignity, privacy, autonomy, kindness, ease of use of the system
Smaranda che	Predictors of a negative labour and birth experience based on a national survey of Canadian women	2016	Measurement	Canada	labor and childbirth	Instrument not validated	communication, ease of use of the system
Dencker	Childbirth experience questionnaire (CEQ): development and evaluation of a multidimensional instrument	2010	Instrument validation	Sweden	labor and childbirth	Validation study	communication, respect & dignity, autonomy, social support, user voice
Turkmen	Post-partum duration of satisfaction with childbirth	2018	Measurement	Sweden	labor and childbirth	Used validated instrument	communication, respect & dignity, autonomy, social support, user voice

Walker	Childbirth experience questionnaire: validating its use in the United Kingdom	2015	Instrument validation	United Kingdom	labor and childbirth	Validation study	communication, respect & dignity, autonomy, social support, kindness, user voice, choice of provider
Bertucci	Assessing the perception of the childbirth experience in Italian women: A contribution to the adaptation of the childbirth perception questionnaire	2012	Instrument validation	Italy	labor and childbirth	Validation study	autonomy, social support
Henderson	Experiencing maternity care: the care received and perceptions of women from different ethnic groups	2013	Measurement	United Kingdom	antenatal, labor and childbirth, postnatal	Instrument not validated	communication, respect & dignity, autonomy, kindness, social support, choice of provider
Redshaw	Women with disability: the experience of maternity care during pregnancy, labour and birth and the postnatal period	2013	Measurement	United Kingdom	antenatal, labor and childbirth, postnatal	Instrument not validated	communication, respect & dignity, autonomy, kindness, social support, choice of provider, user voice
Henderson	Change over time in women's views and experiences of maternity care in England, 1995-2014: A comparison using survey data	2017	Measurement	United Kingdom	antenatal, labor and childbirth, postnatal	Has components of validated instrument	communication, respect & dignity, autonomy, social support
Henderson	Who is well after childbirth? Factors related to positive outcome	2013	Measurement	United Kingdom	antenatal, labor and childbirth, postnatal	Has components of validated instrument	communication, respect & dignity, autonomy, kindness
Kruk	Evaluation Of A Maternal Health Program In Uganda And Zambia Finds Mixed Results On Quality Of Care And Satisfaction	2016	Program/Policy evaluation	Multi-Uganda,Zambia	labor and childbirth	Instrument not validated	communication, respect & dignity, privacy
Larson	Determinants of perceived quality of obstetric care in rural Tanzania: a cross-sectional study	2014	Measurement	Tanzania	labor and childbirth	Instrument not validated	communication, respect & dignity, privacy, affordability
Vedam	Patient-led decision making: Measuring autonomy and respect in Canadian maternity care	2019	Measurement	Canada	antenatal, labor and childbirth, postnatal, newborn	Used validated instrument	communication, respect & dignity, autonomy, user voice, nondiscrimination

Vedam	The Mother's Autonomy in Decision Making (MADM) scale: Patient-led development and psychometric testing of a new instrument to evaluate experience of maternity care	2017	Instrument validation	Canada	antenatal, labor and childbirth, postnatal, newborn	Validation study	communication, respect & dignity, autonomy, user voice
Ford	Are women birthing in New South Wales hospitals satisfied with their care?	2015	Measurement	Australia	labor and childbirth	Used validated instrument	communication, respect & dignity
Todd	"Very Good" Ratings in a Survey of Maternity Care: Kindness and Understanding Matter to Australian Women.	2017	Measurement	Australia	antenatal, labor and childbirth, postnatal	Instrument not validated	communication, kindness, autonomy, social support, user voice, ease of use of the system
Afulani	Predictors of person-centered maternity care: the role of socioeconomic status, empowerment, and facility type	2018	Measurement	Kenya	labor and childbirth	Used validated instrument	communication, respect & dignity, autonomy, confidentiality, social support, wait time
Afulani	Can an integrated obstetric emergency simulation training improve respectful maternity care? Results from a pilot study in Ghana	2019	Program/Policy evaluation	Ghana	labor and childbirth	Used validated instrument	communication, respect & dignity, privacy, autonomy, confidentiality, social support, wait time
Afulani	Development of a tool to measure personcentered maternity care in developing settings: validation in a rural and urban Kenyan population	2017	Instrument validation	Kenya	labor and childbirth	Validation study	communication, respect & dignity, privacy, autonomy, confidentiality, social support, wait time
Afulani	Validation of the person-centered maternity care scale in India	2018	Instrument validation	India	labor and childbirth	Validation study	communication, respect & dignity, privacy, autonomy, confidentiality, social support, ease of use of the system, wait time
Afulani	Person-centred maternity care in low-income and middle-income countries: analysis of data from Kenya, Ghana, and India	2019	Measurement	Multi-Kenya,Ghana,India	labor and childbirth	Used validated instrument	communication, respect & dignity, privacy, autonomy, confidentiality, kindness, social support, wait time

Afulani	Companionship during facility-based childbirth: results from a mixed-methods study with recently delivered women and providers in Kenya	2018	Measurement	Kenya	labor and childbirth	Instrument not validated	social support
Truijens	The Effect of Multiprofessional Simulation-Based Obstetric Team Training on Patient-Reported Quality of Care	2015	Program/Policy evaluation	Netherlands	labor and childbirth	Used validated instrument	communication, respect & dignity, autonomy, confidentiality, social support
Truijens	Development of the Pregnancy and Childbirth Questionnaire (PCQ): evaluating quality of care as perceived by women who recently gave birth	2014	Instrument validation	Netherlands	labor and childbirth	Validation study	communication, respect & dignity, autonomy, confidentiality, kindness, social support
Sjetne	Do experiences with pregnancy, birth and postnatal care in Norway vary by the women's geographic origin? a comparison of cross-sectional survey results	2017	Measurement	Norway	antenatal, labor and childbirth, postnatal	Used validated instrument	communication, respect & dignity, kindness, social support, user voice, choice of provider, ease of use of the system
Sjetne	A questionnaire to measure women's experiences with pregnancy, birth and postnatal care: instrument development and assessment following a national survey in Norway	2015	Instrument validation	Norway	antenatal, labor and childbirth, postnatal	Validation study	communication, respect & dignity, kindness, social support, ease of use of the system
Donate-Manzanares	Cross-cultural adaptation and validation of the psychometric properties of the Quality from the Patient's Perspective I Questionnaire translated into Spanish	2017	Instrument validation	Spain	labor and childbirth	Validation study	communication, respect & dignity, autonomy, kindness, social support, user voice
Gamedze-Mshayisa	Factors associated with women's perception of and satisfaction with quality of intrapartum care practices in Swaziland	2018	Measurement	eSwatini	labor and childbirth	Used validated instrument	communication, respect & dignity, privacy, social support, user voice, wait time

Redshaw	Young women's recent experience of labour and birth care in Queensland	2014	Measurement	Australia	labor and childbirth, newborn	Has components of validated instrument	communication, respect & dignity, privacy, autonomy, kindness, social support
Mander	Perceived Safety, Quality and Cultural Competency of Maternity Care for Culturally and Linguistically Diverse Women in Queensland	2016	Measurement	Australia	antenatal, labor and childbirth, postnatal	Instrument not validated	communication, respect & dignity, privacy, autonomy, kindness, social support, user voice, choice of provider, ease of use of the system
Wyles	Does it get better with age? Women's experience of communication in maternity care	2019	Measurement	Australia	antenatal, labor and childbirth	Instrument not validated	communication, respect & dignity, privacy, autonomy, kindness, user voice
Scheerhag en	Measuring clients' experiences with antenatal care before or after childbirth: it matters	2018	Instrument validation	Netherlands	antenatal, labor and childbirth, postnatal	Used validated instrument	communication, respect & dignity, privacy, autonomy, confidentiality, kindness, social support, user voice, choice of provider, ease of use of the system, wait time
Scheerhag en	Measuring client experiences in maternity care under change: development of a questionnaire based on the WHO Responsiveness model	2015	Instrument validation	Netherlands	antenatal, labor and childbirth, postnatal	Validation study	communication, respect & dignity, privacy, autonomy, confidentiality, kindness, social support, user voice, choice of provider, wait time
Scheerhag en	Applicability of the ReproQ client experiences questionnaire for quality improvement in maternity care	2016	Instrument validation	Netherlands	labor and childbirth	Validation study	communication, respect & dignity, privacy, autonomy, confidentiality, kindness, social support, user voice, choice of provider, wait time
van der Kooy	Quality of perinatal care services from the user's perspective: a Dutch study applies the World Health Organization's responsiveness concept	2017	Measurement	Netherlands	antenatal, labor and childbirth	Used validated instrument	communication, respect & dignity, privacy, autonomy, confidentiality, kindness, social support, user voice, choice of provider, wait time, ease of use of the system

van der Kooy	Validity of a questionnaire measuring the world health organization concept of health system responsiveness with respect to perinatal services in the Dutch obstetric care system	2014	Instrument validation	Netherlands	antenatal, labor and childbirth, postnatal	Validation study	communication, respect & dignity, privacy, autonomy, confidentiality, social support, choice of provider, wait time, ease of use of the system
van Stenus	Client experiences with perinatal healthcare for high-risk and low-risk women	2018	Measurement	Netherlands	labor and childbirth, postnatal	Used validated instrument	communication, respect & dignity, privacy, autonomy, confidentiality, kindness, social support, user voice, wait time, ease of use of the system
Colley	Women's perception of support and control during childbirth in The Gambia, a quantitative study on dignified facility-based intrapartum care	2018	Measurement	Gambia, The	labor and childbirth	Used validated instrument	communication, respect & dignity, privacy, autonomy, social support, kindness, user voice
Ford	Measurement of Maternal Perceptions of Support and Control in Birth (SCIB)	2009	Instrument validation	United Kingdom	labor and childbirth	Validation study	communication, respect & dignity, privacy, autonomy, social support, user voice
Inci	The Turkish version of perceived support and control in birth scale	2015	Instrument validation	Turkey	labor and childbirth	Validation study	communication, respect & dignity, privacy, autonomy, social support, kindness, user voice
Thyagarajan	Parental perceptions of hypothermia treatment for neonatal hypoxic-ischaemic encephalopathy	2018	Measurement	United Kingdom	newborn	Instrument not validated	communication, social support
Abuya	The effect of a multi-component intervention on disrespect and abuse during childbirth in Kenya	2015	Program/Policy evaluation	Kenya	labor and childbirth	Instrument not validated	communication, respect & dignity, privacy, confidentiality, autonomy
Abuya	Exploring the prevalence of disrespect and abuse during childbirth in Kenya	2015	Instrument validation	Kenya	labor and childbirth	Validation study	communication, respect & dignity, privacy, confidentiality, autonomy
Abuya	Measuring mistreatment of women throughout the birthing process: implications for quality of care assessments	2018	Measurement	Kenya	labor and childbirth	Instrument not validated	communication, respect & dignity, privacy, confidentiality, autonomy, kindness

Alzyoud	Exposure to verbal abuse and neglect during childbirth among Jordanian women	2018	Measurement	Jordan	labor and childbirth	Instrument not validated	communication, respect & dignity, privacy, kindness, social support
Anderson	Construct Validity of the Childbirth Trauma Index for Adolescents	2011	Instrument validation	United States	labor and childbirth	Validation study	kindness, social support
Asefa	Prevention of mother-to-child transmission (PMTCT) of HIV services in Adama town, Ethiopia: clients' satisfaction and challenges experienced by service providers	2014	Measurement	Ethiopia	antenatal, other	Instrument not validated	communication, privacy, wait time
Asefa	Status of respectful and non-abusive care during facility-based childbirth in a hospital and health centers in Addis Ababa, Ethiopia	2015	Measurement	Ethiopia	labor and childbirth	Used validated instrument	communication, respect & dignity, autonomy, nondiscrimination, privacy
Ashraf	Assessing women's satisfaction level with maternity services: Evidence from Pakistan	2012	Measurement	Pakistan	labor and childbirth	Has components of validated instrument	communication, wait time
Attanasio	Factors influencing women's perceptions of shared decision making during labor and delivery: Results from a large-scale cohort study of first childbirth	2018	Measurement	United States	labor and childbirth	Instrument not validated	respect & dignity, autonomy, user voice
Attanasio	Patient-reported Communication Quality and Perceived Discrimination in Maternity Care	2015	Measurement	United States	antenatal, labor and childbirth	Instrument not validated	communication, nondiscrimination
Avortri	Predictors of satisfaction with child birth services in public hospitals in Ghana	2011	Measurement	Ghana	labor and childbirth	Has components of validated instrument	communication, respect & dignity, privacy, autonomy, social support, wait time
Azhar	Disrespect and abuse during childbirth in district Gujrat, Pakistan: A quest for respectful maternity care	2018	Measurement	Pakistan	labor and childbirth	Instrument not validated	communication, respect & dignity, privacy, nondiscrimination, social support

Banks	Jeopardizing quality at the frontline of healthcare: prevalence and risk factors for disrespect and abuse during facility-based childbirth in Ethiopia	2018	Measurement	Ethiopia	labor and childbirth	Instrument not validated	communication, respect & dignity, autonomy, privacy, nondiscrimination, confidentiality, kindness
Bashour	The effect of training doctors in communication skills on women's satisfaction with doctor-woman relationship during labour and delivery: A stepped wedge cluster randomised trial in Damascus	2013	Program/Policy evaluation	Syrian Arab Republic	labor and childbirth	Used validated instrument	communication, respect & dignity, kindness
Bernitz	Evaluation of satisfaction with care in a midwifery unit and an obstetric unit: a randomized controlled trial of low-risk women	2016	Program/Policy evaluation	Norway	labor and childbirth	Used validated instrument	ease of use of the system
Bhattachar ya	Silent voices: institutional disrespect and abuse during delivery among women of Varanasi district, northern India	2018	Measurement	India	labor and childbirth	Instrument not validated	communication, respect & dignity, autonomy, privacy, nondiscrimination, confidentiality, social support
Bohren	Methodological development of tools to measure how women are treated during facility-based childbirth in four countries: labor observation and community survey	2018	Measurement	Ghana	labor and childbirth	Used validated instrument	communication, respect & dignity, privacy, nondiscrimination, confidentiality, social support
Brandao	Childbirth experiences related to obstetric violence in public health units in Quito, Ecuador	2018	Measurement	Ecuador	labor and childbirth	Instrument not validated	communication, respect & dignity, autonomy, privacy, nondiscrimination, confidentiality
Colombar a	Institutional Delivery and Satisfaction among Indigenous and Poor Women in Guatemala, Mexico, and Panama	2016	Measurement	Multi-Guat, Mex, Pan	labor and childbirth	Instrument not validated	respect & dignity, autonomy

Creanga	Is quality of care a key predictor of perinatal health care utilization and patient satisfaction in Malawi?	2017	Measurement	Malawi	antenatal, labor and childbirth, postnatal, newborn	Instrument not validated	communication, respect & dignity, privacy, kindness
da Silva	Quality of care for labor and childbirth in a public hospital network in a Brazilian state capital: patient satisfaction	2017	Measurement	Brazil	antenatal, labor and childbirth, newborn	Instrument not validated	communication, respect & dignity, privacy, kindness, social support, user voice, ease of use of the system, wait time
Dauletyaro va	Are Women of East Kazakhstan Satisfied with the Quality of Maternity Care? Implementing the WHO Tool to Assess the Quality of Hospital Services	2016	Measurement	Kazakhstan	antenatal, labor and childbirth, postnatal, newborn	Instrument not validated	communication, respect & dignity, autonomy, social support
Devkota	Do experiences and perceptions about quality of care differ among social groups in Nepal?: A study of maternal healthcare experiences of women with and without disabilities, and Dalit and non-Dalit women	2017	Measurement	Nepal	antenatal, labor and childbirth	Used validated instrument	communication, respect & dignity, privacy, kindness, ease of use of the system
Dey	Discordance in self-report and observation data on mistreatment of women by providers during childbirth in Uttar Pradesh, India	2017	Measurement	India	labor and childbirth	Instrument not validated	communication, respect & dignity, autonomy, nondiscrimination, ease of use of the system
Dynes	Client and provider factors associated with companionship during labor and birth in Kigoma Region, Tanzania	2019	Measurement	Tanzania	labor and childbirth	Instrument not validated	social support
Dynes	Patient and provider determinants for receipt of three dimensions of respectful maternity care in Kigoma Region, TanzaniaApril-July, 2016	2018	Measurement	Tanzania	labor and childbirth	Instrument not validated	communication, respect & dignity, privacy, confidentiality, kindness, social support, user voice, wait time

Feinstein	Antenatal and delivery services in Kinshasa, Democratic Republic of Congo: care-seeking and experiences reported by women in a household-based survey	2013	Measurement of other	Congo, Dem. Rep.	antenatal	Instrument not validated	communication, respect & dignity, privacy, wait time
Fisseha	Quality of the delivery services in health facilities in Northern Ethiopia	2017	Measurement	Ethiopia	labor and childbirth	Instrument not validated	communication
Garrard	Assessing obstetric patient experience: a SERVQUAL questionnaire	2013	Program/Policy evaluation	United Kingdom	antenatal	Has components of validated instrument	communication, respect & dignity, privacy, autonomy, kindness
Gartner	Good reliability and validity for a new utility instrument measuring the birth experience, the Labor and Delivery Index	2015	Instrument validation	Netherlands	labor and childbirth	Validation study	communication, respect & dignity, social support, user voice
Gebremichael	Mothers' experience of disrespect and abuse during maternity care in northern Ethiopia	2018	Measurement	Ethiopia	labor and childbirth	Instrument not validated	communication, respect & dignity, confidentiality, privacy, social support, user voice
Haines	The role of women's attitudinal profiles in satisfaction with the quality of their antenatal and intrapartum care	2013	Measurement	Sweden	antenatal, labor and childbirth	Used validated instrument	communication, autonomy, social support, user voice
Hall	Dignity and respect during pregnancy and childbirth: a survey of the experience of disabled women	2018	Measurement	Multi-UK, Ireland	antenatal, labor and childbirth, postnatal	Instrument not validated	communication, respect & dignity, privacy, nondiscrimination, ease of use of the system
Halperin	A comparison of Israeli Jewish and Arab women's birth perceptions	2014	Measurement	Israel	labor and childbirth	Used validated instrument	communication, respect & dignity, user voice
Hameed	Women's experiences of mistreatment during childbirth: A comparative view of home- and facility-based births in Pakistan	2018	Measurement	Pakistan	labor and childbirth	Instrument not validated	communication, respect & dignity, privacy, nondiscrimination, confidentiality, social support, user voice

Heaman	Quality of prenatal care questionnaire: instrument development and testing	2014	Instrument validation	Canada	antenatal	Validation study	communication, respect & dignity, privacy, autonomy, confidentiality, kindness, social support, user voice, ease of use of the system, wait time
Heatley	Women's Perceptions of Communication in Pregnancy and Childbirth: Influences on Participation and Satisfaction With Care	2015	Measurement	Australia	antenatal, labor and childbirth, postnatal	Instrument not validated	communication, autonomy
Hulton	Applying a framework for assessing the quality of maternal health services in urban India	2007	Measurement	India	labor and childbirth	Instrument not validated	communication, respect & dignity, kindness, ease of use of the system
Igarashi	Immigrants' experiences of maternity care in Japan	2013	Measurement	Japan	antenatal, labor and childbirth, postnatal	Instrument not validated	communication, respect & dignity, kindness
Iida	The relationship between women-centred care and women's birth experiences: A comparison between birth centres, clinics, and hospitals in Japan	2012	Measurement	Japan	labor and childbirth	Used validated instrument	communication, respect & dignity, autonomy, social support
Ijadunola	Lifting the veil on disrespect and abuse in facility-based child birth care: findings from South West Nigeria	2019	Measurement	Nigeria	labor and childbirth	Instrument not validated	communication, respect & dignity, privacy, nondiscrimination, confidentiality, social support
Kambala	Perceptions of quality across the maternal care continuum in the context of a health financing intervention: Evidence from a mixed methods study in rural Malawi	2017	Measurement	Malawi	antenatal, labor and childbirth, postnatal	Instrument not validated	communication, privacy, autonomy, confidentiality, social support, user voice
Karkee	Women's perception of quality of maternity services: a longitudinal survey in Nepal	2014	Measurement	Nepal	labor and childbirth	Has components of validated instrument	respect & dignity, kindness

Kifle	Predictors of Women's Satisfaction with Hospital-Based Intrapartum Care in Asmara Public Hospitals, Eritrea	2017	Measurement	Eritrea	labor and childbirth	Instrument not validated	communication, respect & dignity, privacy, autonomy, social support, ease of use of the system
Kigenyi	Quality of intrapartum care at Mulago national referral hospital, Uganda: clients' perspective	2013	Measurement	Uganda	labor and childbirth	Instrument not validated	respect & dignity, privacy, autonomy, confidentiality, wait time
Lacaze-Masmonteil	Perception du contexte linguistique et culturel minoritaire sur le vÃ©cu de la grossesse	2013	Measurement	Canada	antenatal, labor and childbirth, postnatal	Instrument not validated	communication, social support, ease of use of the system, wait time
Lee	Efficacy ofWarm Showers on Labor Pain and Birth Experiences During the First Labor Stage	2013	Program/Policy evaluation	Taiwan	labor and childbirth	Used validated instrument	autonomy, social support
Lewis	Development and validation of a measure of informed choice for women undergoing non-invasive prenatal testing for aneuploidy	2016	Instrument validation	United Kingdom	antenatal	Validation study	autonomy
Liabsuetrakul	Health system responsiveness for delivery care in Southern Thailand	2012	Measurement	Thailand	labor and childbirth	Instrument not validated	communication, respect & dignity, autonomy, social support, choice of provider
Lin	Comparison between pregnant Southeast Asian immigrant and Taiwanese women in terms of pregnancy knowledge, attitude toward pregnancy, medical service experiences and prenatal care behaviors	2008	Measurement	Taiwan	antenatal	Instrument not validated	communication, ease of use of the system
Lindquist	Experiences, utilisation and outcomes of maternity care in England among women from different socio-economic groups: findings from the 2010 National Maternity Survey	2015	Measurement	United Kingdom	antenatal, labor and childbirth, postnatal	Instrument not validated	communication, respect & dignity, autonomy, ease of use of the system

Macfarlane	Survey of women's experiences of care in a new freestanding midwifery unit in an inner city area of London, England. 1: Methods and women's overall ratings of care	2014	Program/Policy evaluation	United Kingdom	antenatal, labor and childbirth, postnatal	Instrument not validated	respect & dignity, privacy
Macfarlane	Survey of women's experiences of care in a new freestanding midwifery unit in an inner city area of London, England: 2. Specific aspects of care	2014	Measurement	United Kingdom	labor and childbirth	Instrument not validated	communication, respect & dignity
Mahar	Quantity and quality of information, education and communication during antenatal visit at private and public sector hospitals of Bahawalpur, Pakistan	2012	Measurement	Pakistan	antenatal	Has components of validated instrument	communication
Malouf	Access and quality of maternity care for disabled women during pregnancy, birth and the postnatal period in England: data from a national survey	2017	Measurement	United Kingdom	abortion, antenatal, labor and childbirth, postnatal, newborn	Instrument not validated	communication, respect & dignity, kindness, social support, user voice, ease of use of the system, wait time
Mannarini	A Rasch-based dimension of delivery experience: spontaneous vs. medically assisted conception	2013	Measurement	Italy	labor and childbirth	Instrument not validated	respect & dignity
Martin	Midwives' perceptions of communication during videotaped counseling for prenatal anomaly tests: how do they relate to clients' perceptions and independent observations?	2015	Instrument validation	Netherlands	antenatal	Validation study	communication, autonomy
McLachlan	A randomised controlled trial of caseload midwifery for women at low risk of medical complications (COSMOS): Women's satisfaction with care	2012	Program/Policy evaluation	Australia	antenatal, labor and childbirth, postnatal	Instrument not validated	communication, respect & dignity, privacy, autonomy, kindness, social support, user voice

Mohammad	Jordanian women's dissatisfaction with childbirth care	2013	Measurement	Jordan	labor and childbirth	Used validated instrument	communication, respect & dignity, privacy, autonomy, kindness, user voice
Molina	Delivery practices and care experience during implementation of an adapted safe childbirth checklist and respectful care program in Chiapas, Mexico	2019	Measurement	Mexico	labor and childbirth	Instrument not validated	communication, privacy, social support
Molloy	Improving practice: women's views of a maternity triage service	2010	Measurement	United Kingdom	other	Instrument not validated	communication, respect & dignity, wait time
Montesinos-Segura	Disrespect and abuse during childbirth in fourteen hospitals in nine cities of Peru	2017	Measurement	Peru	labor and childbirth	Instrument not validated	communication, respect & dignity, privacy, nondiscrimination, confidentiality, social support
Moosavisadat	Comparison of maternity care quality in teaching and non-teaching hospitals in Khorram Abad, Islamic Republic of Iran	2011	Measurement	Iran, Islamic Rep.	labor and childbirth, postnatal, newborn	Used validated instrument	communication, privacy
Mukamurigo	Associations between perceptions of care and women's childbirth experience: a population-based cross-sectional study in Rwanda	2017	Measurement	Rwanda	labor and childbirth	Has components of validated instrument	communication, respect & dignity, social support
Mulherin	Weight stigma in maternity care: women's experiences and care providers' attitudes	2013	Measurement	Australia	antenatal, labor and childbirth, postnatal	Instrument not validated	communication, respect & dignity, privacy, kindness, social support
Mutaganzwa	Advancing the health of women and newborns: predictors of patient satisfaction among women attending antenatal and maternity care in rural Rwanda	2018	Measurement	Rwanda	antenatal, labor and childbirth	Instrument not validated	communication, respect & dignity, privacy, confidentiality, affordability, ease of use of the system, wait time
Na	An early stage evaluation of the Supporting Program for Obstetric Care Underserved Areas in	2014	Measurement	Korea, Rep.	labor and childbirth	Instrument not validated	kindness

	Korea						
Nababan	Improving quality of care for maternal and newborn health: a pre-post evaluation of the Safe Childbirth Checklist at a hospital in Bangladesh	2017	Program/Policy evaluation	Bangladesh	labor and childbirth	Has components of validated instrument	communication, social support
Nnebue	Clients' knowledge, perception and satisfaction with quality of maternal health care services at the primary health care level in Nnewi, Nigeria	2014	Measurement	Nigeria	antenatal, labor and childbirth, postnatal, other	Instrument not validated	wait time
Oikawa	Assessment of maternal satisfaction with facility-based childbirth care in the rural region of Tambacouda, Senegal	2014	Measurement	Senegal	labor and childbirth	Instrument not validated	communication, privacy, kindness, social support
Okafor	Disrespect and abuse during facility-based childbirth in a low-income country	2015	Measurement	Nigeria	labor and childbirth	Used validated instrument	communication, respect & dignity, nondiscrimination, autonomy, confidentiality, privacy, social support
Oladapo	Quality of antenatal services at the primary care level in southwest Nigeria	2008	Measurement	Nigeria	antenatal	Has components of validated instrument	communication, respect & dignity, privacy, nondiscrimination, autonomy, kindness, social support, wait time
Onyeajam	Antenatal care satisfaction in a developing country: a cross-sectional study from Nigeria	2018	Measurement	Nigeria	antenatal	Instrument not validated	communication, respect & dignity, privacy, nondiscrimination, kindness, affordability, wait time
Oskay	Evaluation of Patients' Satisfaction With Nursing Students' Care on a Perinatology Ward	2015	Measurement	Turkey	antenatal, labor and childbirth, other	Used validated instrument	communication, respect & dignity, kindness, social support
Overgaard	The impact of birthplace on women's birth experiences and perceptions of care	2012	Measurement	Denmark	labor and childbirth, postnatal	Instrument not validated	communication, respect & dignity, autonomy, social support, user voice

Oweis	Jordanian mother's report of their childbirth experience: findings from a questionnaire survey	2009	Measurement	Jordan	labor and childbirth	Used validated instrument	communication, respect & dignity, privacy, nondiscrimination, autonomy, social support, ease of use of the system
Paul	Improving satisfaction with care and reducing length of stay in an obstetric triage unit using a nurse-midwife-managed model of care	2013	Measurement	United States	other	Has components of validated instrument	communication, wait time
Phaladi-Digamela	Community-physician-based versus hospital-based antenatal care: A comparison of patient satisfaction	2014	Measurement	South Africa	antenatal	Used validated instrument	communication, respect & dignity, user voice, wait time
Pinidiyapa thirage	Antenatal care provided and its quality in field clinics in Gampaha District, Sri Lanka	2007	Measurement	Sri Lanka	antenatal	Instrument not validated	communication
Qureshi	Patient satisfaction at tertiary care hospitals in Kashmir: a study from the Lala Ded Hospital Kashmir India	2009	Measurement	India	other	Instrument not validated	communication, ease of use of the system
Rabbani	Service quality in contracted facilities	2015	Measurement	Pakistan	antenatal	Has components of validated instrument	communication
Raj	Associations Between Mistreatment by a Provider during Childbirth and Maternal Health Complications in Uttar Pradesh, India	2017	Measurement	India	labor and childbirth	Instrument not validated	communication, respect & dignity, autonomy, nondiscrimination
Raleigh	Ethnic and social inequalities in women's experience of maternity care in England: results of a national survey	2010	Measurement	United Kingdom	antenatal, labor and childbirth, postnatal	Has components of validated instrument	communication, respect & dignity, autonomy, social support, choice of provider, ease of use of the system
Ratcliffe	Mitigating disrespect and abuse during childbirth in Tanzania: an exploratory study of the effects of two facility-based interventions in a large public hospital	2016	Measurement	Tanzania	labor and childbirth	Has components of validated instrument	respect & dignity

Redshaw	Validation of a perceptions of care adjective checklist	2009	Instrument validation	United Kingdom	labor and childbirth	Validation study	communication, respect & dignity, kindness, social support
Ribeiro	CONTENTMENT OF PUERPERAL WOMEN ASSISTED BY OBSTETRIC NURSES	2018	Measurement	Brazil	labor and childbirth	Instrument not validated	communication, respect & dignity, kindness, social support
Robertson	Comparison of centering pregnancy to traditional care in Hispanic mothers	2009	Measurement	United States	antenatal	Has components of validated instrument	user voice
Roosevelt	Psychometric assessment of the Health Care Alliance Questionnaire with women in prenatal care	2015	Instrument validation	United States	antenatal	Validation study	communication, respect & dignity, confidentiality, autonomy, kindness, social support
Rubashkin	Assessing quality of maternity care in Hungary: expert validation and testing of the mother-centered prenatal care (MCPC) survey instrument	2017	Instrument validation	Hungary	antenatal, labor and childbirth, postnatal	Validation study	communication, respect & dignity, nondiscrimination, autonomy, user voice, affordability, choice of provider
Rudman	Evaluating multi-dimensional aspects of postnatal hospital care	2008	Measurement	Sweden	postnatal	Instrument not validated	communication, respect & dignity, kindness
Sabanayagam	Attitudes and perceptions of pregnant women with CHD: results of a single-site survey	2017	Measurement	United States	antenatal, postnatal	Instrument not validated	communication, choice of provider
Saggurti	Effect of health intervention integration within womenâ©ɷs self-help groups on collectivization and healthy practices around reproductive, maternal, neonatal and child health in rural India	2018	Program/Policy evaluation	India	labor and childbirth	Instrument not validated	social support
Saima	Assessing patient satisfaction in gynaecology and obstetrics in tertiary care hospital	2015	Measurement	Pakistan	other	Has components of validated instrument	communication, respect & dignity, nondiscrimination, autonomy

Saizonou	Quality Assessment of Refocused Antenatal Care Services at the District Hospital of Suru-Léré in Benin	2014	Measurement	Benin	antenatal	Instrument not validated	communication, respect & dignity, affordability, ease of use of the system
Sapountzi-Krepia	Mothers' experiences of maternity services: internal consistency and test-retest reliability of the Greek translation of the Kuopio Instrument for Mothers	2009	Instrument validation	Greece	labor and childbirth	Validation study	communication, respect & dignity, autonomy
Sawyer	Measuring parents' experiences and satisfaction with care during very preterm birth: a questionnaire development study	2014	Instrument validation	United Kingdom	labor and childbirth	Validation study	communication, kindness, social support, user voice
Sebastian	Associations Between Maternity Care Practices and 2-Month Breastfeeding Duration Vary by Race, Ethnicity, and Acculturation	2019	Measurement	Mexico	labor and childbirth	Instrument not validated	communication, ease of use of the system
Sethi	The prevalence of disrespect and abuse during facility-based maternity care in Malawi: evidence from direct observations of labor and delivery	2017	Measurement	Malawi	labor and childbirth	Instrument not validated	communication, respect & dignity, privacy, social support, user voice
Sharma	An investigation into mistreatment of women during labour and childbirth in maternity care facilities in Uttar Pradesh, India: a mixed methods study	2019	Measurement	India	labor and childbirth	Instrument not validated	communication, respect & dignity, privacy, social support
Sheferaw	Development of a tool to measure women's perception of respectful maternity care in public health facilities	2016	Instrument validation	Ethiopia	labor and childbirth	Validation study	communication, respect & dignity, autonomy, privacy, social support, nondiscrimination, kindness, wait time
Shferaw	Respectful maternity care in Ethiopian public health facilities	2017	Measurement	Ethiopia	labor and childbirth	Instrument not validated	communication, respect & dignity, privacy, social support, user voice

Shimizu	Maternal perceptions of family-centred support and their associations with the mother-nurse relationship in the neonatal intensive care unit	2018	Measurement	Japan	postnatal, newborn	Used validated instrument	communication, respect & dignity, autonomy, social support, user voice, ease of use of the system
Sholeye	Client perception of antenatal care services at primary health centers in an urban area of Lagos, Nigeria	2013	Measurement	Nigeria	antenatal	Instrument not validated	communication, autonomy
Siassakos	A simple tool to measure patient perceptions of operative birth	2009	Instrument validation	United Kingdom	labor and childbirth	Validation study	communication, respect & dignity
Sigurdard ottir	The predictive role of support in the birth experience: A longitudinal cohort study	2017	Measurement	Iceland	antenatal, labor and childbirth, postnatal	Instrument not validated	social support
Soheily	A Comparative Study of Satisfaction of Midwives and Mothers of Adherence to Patient Rights	2017	Measurement	Iran, Islamic Rep.	antenatal, labor and childbirth	Instrument not validated	communication, privacy, autonomy, user voice
Spira	Improving the quality of maternity services in Nepal through accelerated implementation of essential interventions by healthcare professional associations	2018	Measurement of other	Nepal	labor and childbirth	Instrument not validated	social support
Stojanovs ki	The Influence of Ethnicity and Displacement on Quality of Antenatal Care: The Case of Roma, Ashkali, and Balkan Egyptian Communities in Kosovo	2017	Measurement	Kosovo	antenatal	Instrument not validated	communication
Sword	Quality of prenatal care questionnaire: psychometric testing in an Australia population	2015	Instrument validation	Australia	antenatal	Validation study	communication, respect & dignity, autonomy, kindness, social support
Takacs	Social psychological predictors of satisfaction with intrapartum and postpartum care - what matters to women in Czech maternity	2015	Measurement	Czech Republic	labor and childbirth, postnatal	Used validated instrument	communication, respect & dignity, autonomy

hospitals?

Tan	Investigating factors associated with success of breastfeeding in first-time mothers undergoing epidural analgesia: a prospective cohort study	2018	Measurement of other	Singapore	labor and childbirth	Instrument not validated	social support
Tancred	Using mixed methods to evaluate perceived quality of care in southern Tanzania	2016	Measurement	Tanzania	labor and childbirth	Instrument not validated	respect & dignity, social support
Tocchioni	Socio-demographic determinants of women's satisfaction with prenatal and delivery care services in Italy	2018	Measurement	Italy	antenatal, labor and childbirth	Instrument not validated	communication, social support
Tomlinson	Improved management of stillbirth using a care pathway	2018	Program/Policy evaluation	United Kingdom	labor and childbirth, postnatal	Instrument not validated	communication, respect & dignity, social support, user voice
Tougher	Effect of a multifaceted social franchising model on quality and coverage of maternal, newborn, and reproductive health-care services in Uttar Pradesh, India: a quasi-experimental study	2018	Program/Policy evaluation	India	antenatal, labor and childbirth	Instrument not validated	communication, respect & dignity, autonomy, privacy, nondiscrimination, social support, affordability
Truijens	Development of the Childbirth Perception Scale (CPS): perception of delivery and the first postpartum week	2014	Instrument validation	Netherlands	labor and childbirth, postnatal	Validation study	respect & dignity, social support
Ulfsdottir	The association between labour variables and primiparous women's experience of childbirth; a prospective cohort study	2014	Measurement	Sweden	labor and childbirth	Instrument not validated	social support
Uludag	Development and Testing of Women's Perception for the Scale of Supportive Care Given During Labor	2015	Instrument validation	Turkey	labor and childbirth	Validation study	communication, respect & dignity, privacy, kindness, social support, user voice

Vedam	The Mothers on Respect (MOR) index: measuring quality, safety, and human rights in childbirth	2017	Instrument validation	Canada	antenatal, labor and childbirth, postnatal	Validation study	communication, respect & dignity, nondiscrimination, autonomy, choice of provider
Vinturache	Recall of Prenatal Counselling Among Obese and Overweight Women from a Canadian Population: A Population Based Study	2017	Instrument validation	Canada	antenatal	Instrument not validated	communication, user voice
Wang	Perceived Needs of Parents of Premature Infants in NICU	2018	Measurement	China	postnatal, newborn	Used validated instrument	communication, respect & dignity, social support, ease of use of the system
Wassihun	Prevalence of disrespect and abuse of women during child birth and associated factors in Bahir Dar town, Ethiopia	2018	Measurement	Ethiopia	labor and childbirth	Instrument not validated	communication, respect & dignity, autonomy, privacy, nondiscrimination, confidentiality, social support, user voice
Wassihun	Compassionate and respectful maternity care during facility based child birth and womenâ⓪ஃs intent to use maternity service in Bahir Dar, Ethiopia	2018	Measurement	Ethiopia	labor and childbirth	Instrument not validated	communication, respect & dignity, nondiscrimination, confidentiality, kindness, wait time
Wesson	Provider and client perspectives on maternity care in Namibia: results from two cross-sectional studies	2018	Measurement	Namibia	labor and childbirth	Instrument not validated	communication, respect & dignity, privacy, nondiscrimination, confidentiality, social support, affordability, ease of use of the system
Wiegers	The quality of maternity care services as experienced by women in the Netherlands	2009	Measurement	Netherlands	antenatal, labor and childbirth, postnatal	Used validated instrument	communication, respect & dignity, user voice
Ziabakhsh	Voices of Postpartum Women: Exploring Canadian Women’s Experiences of Inpatient Postpartum Care	2018	Measurement	Canada	postnatal, newborn	Instrument not validated	communication, kindness, social support, user voice
